# Analysis of the value of folk music intangible cultural heritage on the regulation of mental health

**DOI:** 10.3389/fpsyt.2023.1067753

**Published:** 2023-03-29

**Authors:** Hui Ning

**Affiliations:** The College of Humanities and Economic Management, Xian Traffic Engineering Institute, Xian, Shaanxi, China

**Keywords:** folk music, safeguarding of intangible cultural heritage, mental health, music therapy, questionnaire survey

## Abstract

This paper proposes an approach based on the safeguarding of intangible cultural heritage (ICH) by studying the value of ICH to explore the value of the ICH of folk music on mental health and its safeguarding measures. Additionally, a questionnaire survey is conducted on the value of the ICH of folk music among college students. The Tibetan Guozhuang dance and music in the ICH are taken as the object. The students’ awareness, participation, and effects on physical and mental health, emotional regulation, and stress regulation are investigated, to study the safeguarding value of folk music. The survey results reveal that in the process of participating in the folk art of Tibetan Guozhuang dance, 41.8% of the students consider it very useful for emotional regulation and stress relief, and 46.31% believe it is useful. 36.95% of the students feel that it is very useful for the development of mental health, and 49.75% think it is useful. This indicates that a total of 86.7% of the students believe that the dance is helpful to the development of students’ mental health. And most of the students are in a happy mood when participating in the dance. Among them, 71.7% of the students say that they are elated, and 66.98% feel that they are excited. It illustrates that as a young group, the students are fond of folk art, but they lack the cognitive approach. Finally, the safeguarding suggestions and implementation paths are put forward in view of the existing problems of the ICH of folk music. The research can provide a research reference for the safeguarding of the ICH of folk music.

## 1. Introduction

The transmission of intangible cultural heritage (ICH) lies with the inheritors. Its benign development can promote local economic development. With economic income, the number of inheritors will increase, and ICH items will be better passed on (1–3). Rational and effective development and utilization of ICH also contribute to the positive interaction between culture and economy (4–6). Taking the ICH of folk music as an example, their special manifestations, unique culture, and national historical spirit are the cultural treasures of mankind. But at present, valuable folk music have been lost, and most of the existing inheritors of skills are elderly, which is not conducive to the inheritance of culture (7–9). An effective way to promote this special form of folk music culture can promote traditional culture. Also, the music itself has great value for people’s mental health (10–12). The emotional performance of music itself has a certain or even huge impact on psychology, and also has a significant effect on the development of children’s intelligence and emotional control. Furthermore, psychologists and psychiatrists now use music therapy on many patients who need emotional and psychological treatment. Comprehensive consideration, the inheritance and safeguarding of the ICH of folk music has important implementation significance.

Music therapy experts generally believe that music therapy affects people’s emotions through music-specific signals. It regulates and improves the physiological functions of human organs through the cerebral cortex, hypothalamus, and limbic system. Thus, music can enhance the internal stability of the body, relieve the adverse physical reactions caused by stimulation, regulate the imbalance of psychology, and restore the normal state of the human body functions (13–16). The famous American psychologist Arnold believes that if a person’s emotions have problems, there must be some irrational ideas in his mind (15). If this irrationality is corrected, the emotional problem will be solved. Traditional psychotherapy believes that cognition determines emotions, while music psychotherapy believes that emotions determine cognition. The influence of music on people’s emotions is very huge. The quality of a person’s emotions largely determines his attitude toward the things around him (17–19). However, if general music therapy wants to get better therapeutic results, it is also necessary to consider many aspects, such as music repertoire, rhythm, and therapeutic environment (20). Chinese folk music mostly comes from the folk. It has great ethnic characteristics and is different from general music, so studying its value in alleviating mental health problems is of great significance to improve the therapeutic effect of music therapy (21–23).

Literature research and questionnaire surveys are used. The value of ICH is studied. The research subjects are Tibetan Guozhuang dance and music. Then, students’ awareness and participation in ICH, such as folk music and dance, and the value and significance of such folk art to students’ physical and mental health, emotional regulation, and stress relief, are investigated. Finally, the solutions to the current problems of the ICH of folk music and dance are given, which can provide new ideas for the research of special music treatment for mental health problems.

## 2. Literature review

Folk music is a unique part of ICH. For the value of folk music to human physical and mental health, scholars from all walks of life have also carried out experiments and investigations in different directions. Su (24) noted that the tension between safeguarding and commercialization of ICH would intensify when it was seen as a heritage in need of safeguarding and a commodified resource. Masoud et al. (25) studied the ICH of the city of Esfahan (Iran) as a new tourist attraction. In addition, the study had three main objectives. The first was to investigate tourists’ inclination toward intangible heritage; the second was to measure visitors’ awareness of intangible heritage; the third was to prioritize intangible heritage attractions and activities from the perspective of domestic tourists. Beck (26) reflected the originality of the songbook brought out from the remote areas of the Sothern Jharkhand plateau as the country’s rich ICH in the study. Su (27) illustrated the birth of ICH in China by analyzing the origin, discourse, and practice of the concept of the authenticity of ICH and the difficulties arising from this concept. It mediated the intermediary between local and international ideologies, elaborated on the history of the complex relationship between authenticity and ICH over the past 20 years, and revealed the difficulties of integrating authenticity and ICH as official discourses. The intelligent environment developed with the support of a new generation of cyber-physical systems can achieve a high concentration of information resources (28). The unique feature extraction methods of deep learning methods can still play an important role in the research of various fields (29).

Music clearly plays an important role in the way the human body heals. First, Perrot (30) combined all sources of famous and minor sanctuaries. Second, some new perspectives on the specificity of Asclepian soundscapes were offered. Elpus and Abril (31) demonstrated the healing methods in makams that were still in use from the 9th century to the present day, illustrating not only the old sheet music but also the new one that followed the tradition of music writing. Frequencies through specific tones were shown to be effective in treating psychological problems. This idea was linked to musical knowledge and mathematical calculations from the 11th century to the present day. This new perspective had a clear answer to the relationship between hospital architecture and music therapy to treat psychological problems. Abrams (32) stated that music therapy, in all its various manifestations, was influenced by fundamental relationships with others within human beings. Therefore, attention needed to be paid to the relationship between music therapy and humanism. Vaudreuil et al. (33) found that participants responded positively to music therapy and community music engagement through telehealth and reported reductions in pain, anxiety, and depression combined with discussions of clinical, ethical, and technical considerations and research related to telemedicine for music therapy through experience teaching and daily application of music. This finding confirmed the role of remote music therapy in the treatment of mental illness. Sharda et al. (34) re-examined the link between music and autism. In addition to qualitative and empirical views, the experience and evidence about the effect of music therapy on autistic children now only exist in medical journals.

Therefore, the safeguarding of the ICH of folk art is currently facing a great crisis, and effective safeguarding measures are needed to carry out cultural inheritance. There are few studies on the role of folk music in mental health treatment, and the findings and measures here can fill this research gap.

## 3. Materials and methods

### 3.1. Safeguarding of ICH

**Figure 1:**
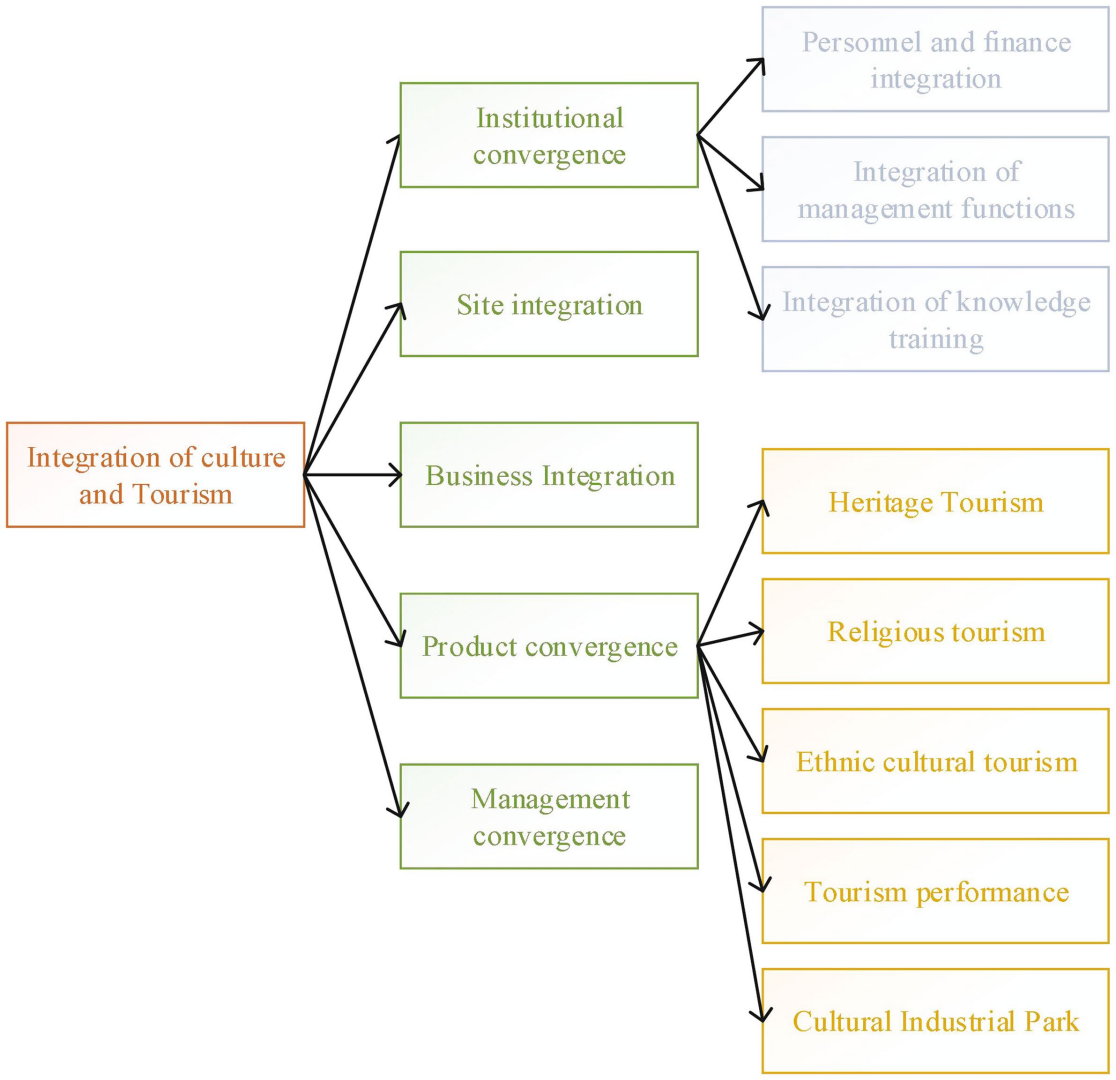
The way of integration of culture and tourism.

ICH is the oldest and most vivid cultural and historical tradition, an important resource and library of the soft power of national and national culture, and an organic composition and important symbol of national characteristics, spirit, emotion, history, personality, temperament, cohesion, and centripetal force (35–37). The recognition, safeguarding, inheritance, and promotion of the nature of ICH play an important role in promoting the economy and building a socialist core value system (38–40). In the future, on the road to cultural and tourism integration, it is necessary to achieve the integration of five major aspects, namely, the integration of institutions, places, business formats, products, and management. This is similar to the study by Jørgensen and McKercher (2), as shown in Figure 1.

Intangible cultural heritage is seldom used for tourism project development, because of the fear of combining ICH with tourism, it will be damaged, which is not conducive to inheritance and safeguarding (41). There are indeed reasons for this, but the safeguarding and development of it is a dialectical and unified relationship. Let Chinese culture go out, and let the world culture come in. In this way, cultural exchanges and communication can be carried out better. At the same time, it is also conducive to enhancing the cultural confidence of the people. Scientific tourism development is an effective way to safeguard ICH, which can realize the interactive development of tourism and culture, culture, and economy. The development of the tourism economy can drive the promotion of the value of ICH. The transformation of it from traditional sightseeing tours to experience tourism and leisure tourism shows that the demand for cultural tourism has become the core of tourism demand. The unique cultural value of ICH just meets this demand. Folk music is like a living organism, it has a natural metabolism, that is, with the passage of time, changes, adjustments, disappearances, and derivations of art forms are bound to occur. The safeguarding of folk music and the inheritance of new forms can not only help the classics persist, but also reflect the social and cultural standards and national soul.

When the material of folk music enters the works of the composer, it has also been transferred to the soul and essence of art, which has a unique effect on people’s spiritual world and mental health. Maybe in the future, the folk music varieties that people can see now will disappear, but as long as these works with their artistic essence and soul exist, it means that they have not really disappeared. While some ancient folk arts are gradually disappearing, new art forms are constantly being derived, and these new art forms invisibly inherit the seemingly disappeared folk music. In the face of such a new generation of folk literature and art, it has to be said that folk music seems to have its own adjustment system. With the change of the external environment, it is also consciously or unconsciously completing its own transformation. For these emerging folk literature and art, as well as the previously mentioned new forms of interpretation and expression of traditional folk music works, there have been mixed comments in the community. But time is the ultimate criterion for testing works of art. For the evaluation of many things, people need to wait patiently, and should show a tolerant attitude. There is no need to rush to conclusions, but they must be given enough attention.

### 3.2. Investigation on the influence of folk music intangible culture on mental health

**Figure 2:**
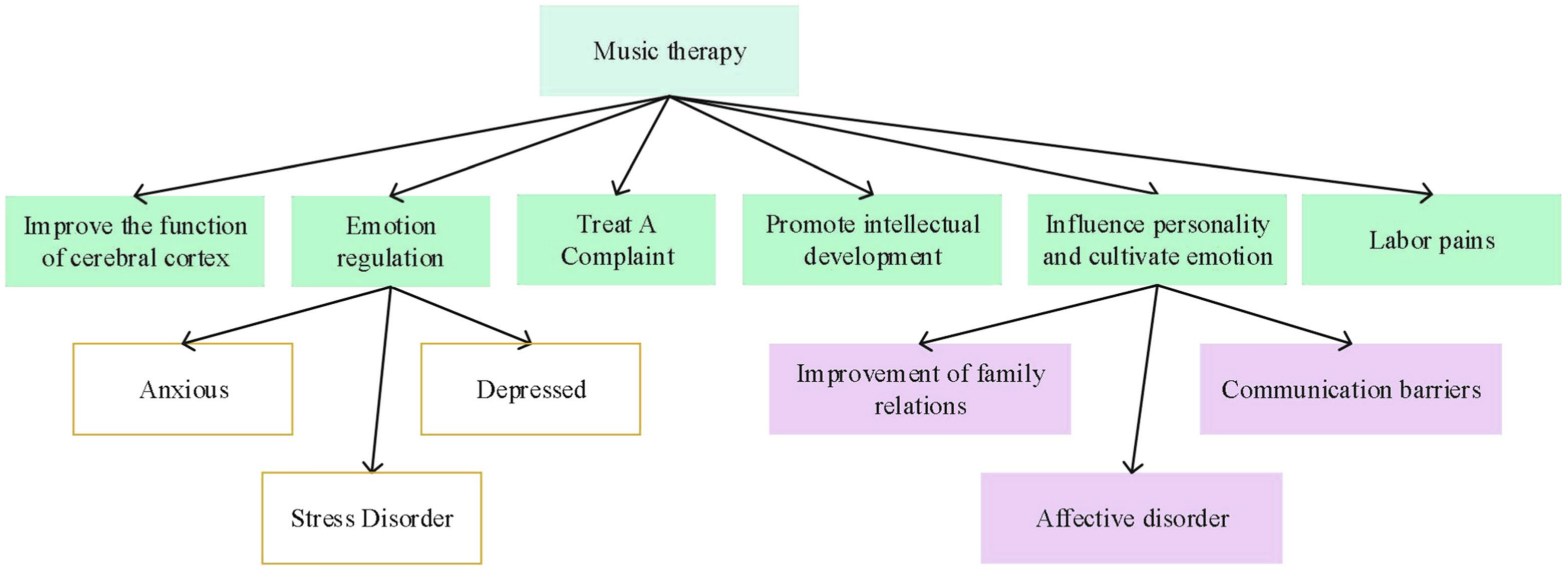
Effects of music therapy on physical and mental health.

Music is an art form that can have a huge impact on the human brain and mind. To a certain extent, it will shape human cognition and subtly affect behavior. The ultimate purpose of music is to enable people to acquire a certain emotional experience, which in turn depends on the cognition of the various components of music. The gradual change of mood brings infinite reverie. If a person is addicted to the emotions of music, he will be carried away by the music and become the protagonist or even the performer in the music story, releasing his body and mind in the process. The effect of music on mental health is displayed in Figure 2 (13).

As Figure 2 suggests, music therapy has a clear alleviating effect on people’s physical health and mental health problems. Music therapy is based on scientific research and is widely used in clinical psychological interventions. It is often considered a type of expressive therapy. In the course of psychological intervention, music therapists use music and its physical, emotional, psychological, social, esthetic, and spiritual roles to help clients improve and maintain their health.

Folk music has strong variability in both content and form because of its special formation method, mainly the collective creation of the people, and relying on oral transmission. As one of the ICHs, Tibetan Guozhuang dance originally meant dancing around a stone pot. Tibetan means people form a circle to sing and dance. It is one of the three major Tibetan folk dances. It is distributed in Qamdo and Nagqu in Tibet, Aba and Ganzi in Sichuan, Diqing in Yunnan, and Tibetan areas in Qinghai and Gansu. During the dance, the men and women are usually lined up in a semicircle and hold hands to form a circle. One person takes the lead, questions and answers are divided between men and women, and they sing duets repeatedly, without musical instrument accompaniment. The dances by young people belong to the “new-style Guozhuang,” with music accompaniment and no need to sing by themselves. The music and meaning of this dance are varied and very rich. The distinctive feature of the music is that the melody is concise and the rhythm is clear. It is dominated by strumming, tapping, and slapping, and it sounds rhythmic. The music is cheerful and heroic. The dance contains the unique charm of Tibetan style, with a strong vitality and artistic, cultural, and historical value.

Because this dance is a collective form of ethnic dance, it gradually appeared in the inter-class exercises in universities for nationalities. To investigate the impact of Tibetan Guozhuang dance and folk music accompanying the dance on the lives and psychological conditions of students in a university for nationalities, it conducted a questionnaire survey on students in an ethnic college in October 2021. The design of the questionnaire is improved based on the music response evaluation scale designed by Nordoffand Robbin (42). The content of the questionnaire is divided into four parts, with a total of 25 questions, mainly focusing on the impact of Guozhuang dance and music on the mental health of ethnic college students. The first part is to investigate the basic situation of participating students (5 questions), and the second part is to study the understanding of this dance among middle school students of this ethnic group (5 questions). The third part is to investigate the students’ views on the influence of this dance on fitness, mental health, national cultural heritage, and extracurricular life (10 questions). The fourth part is a suggestion (5 questions) for the development of dance and music in a university.

The questionnaire survey is carried out randomly in universities for nationality, and a total of 500 questionnaires are distributed, excluding 14 incorrect and incomplete questionnaires, and finally, 486 valid questionnaires are recovered. The reliability and validity of the designed questionnaire are analyzed, and the Kaiser-Meyer-Olkin (KMO) value is used for validity analysis. The KMO is 0.873 > 0.7, indicating that the validity of the questionnaire is good. The Cronbach α coefficients of the reliability indicators of the questionnaire items of different dimensions are all above 0.8, illustrating that the reliability of the questionnaire is good, the quality of the questionnaire is up to the standard, and the survey can be carried out.

## 4. Results and discussion

### 4.1. Results of a survey on the value impact of Guozhuang dance and music on college students

**Figure 3:**
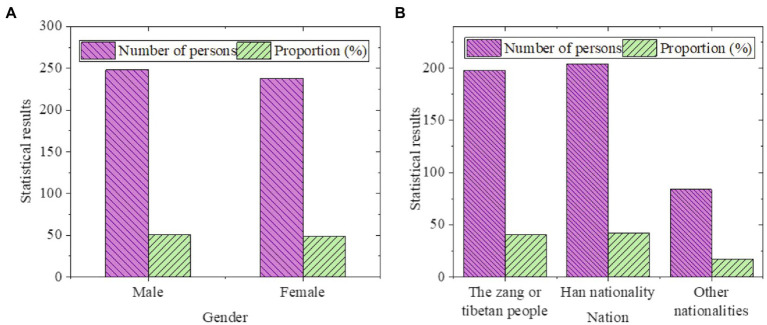
Statistical results of basic information of survey respondents (**A**: by gender; **B**: by educational background).

**Figure 4:**
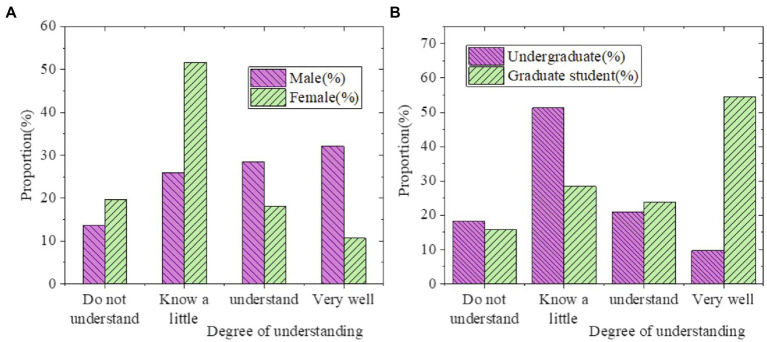
University students’ understanding of Guozhuang dance (**A**: Statistics by gender; **B**: Statistics by educational background).

Figure 3 reveals the basic situation of the surveyed respondents:

In Figure 3, the proportion of males and females participating in this survey is almost equal, with almost half of the students. Most of the students are Han and Tibetan, and a few students are other minorities. The statistical results of the popularity of Guozhuang dance among college students are displayed in Figure 4.

**Figure 5:**
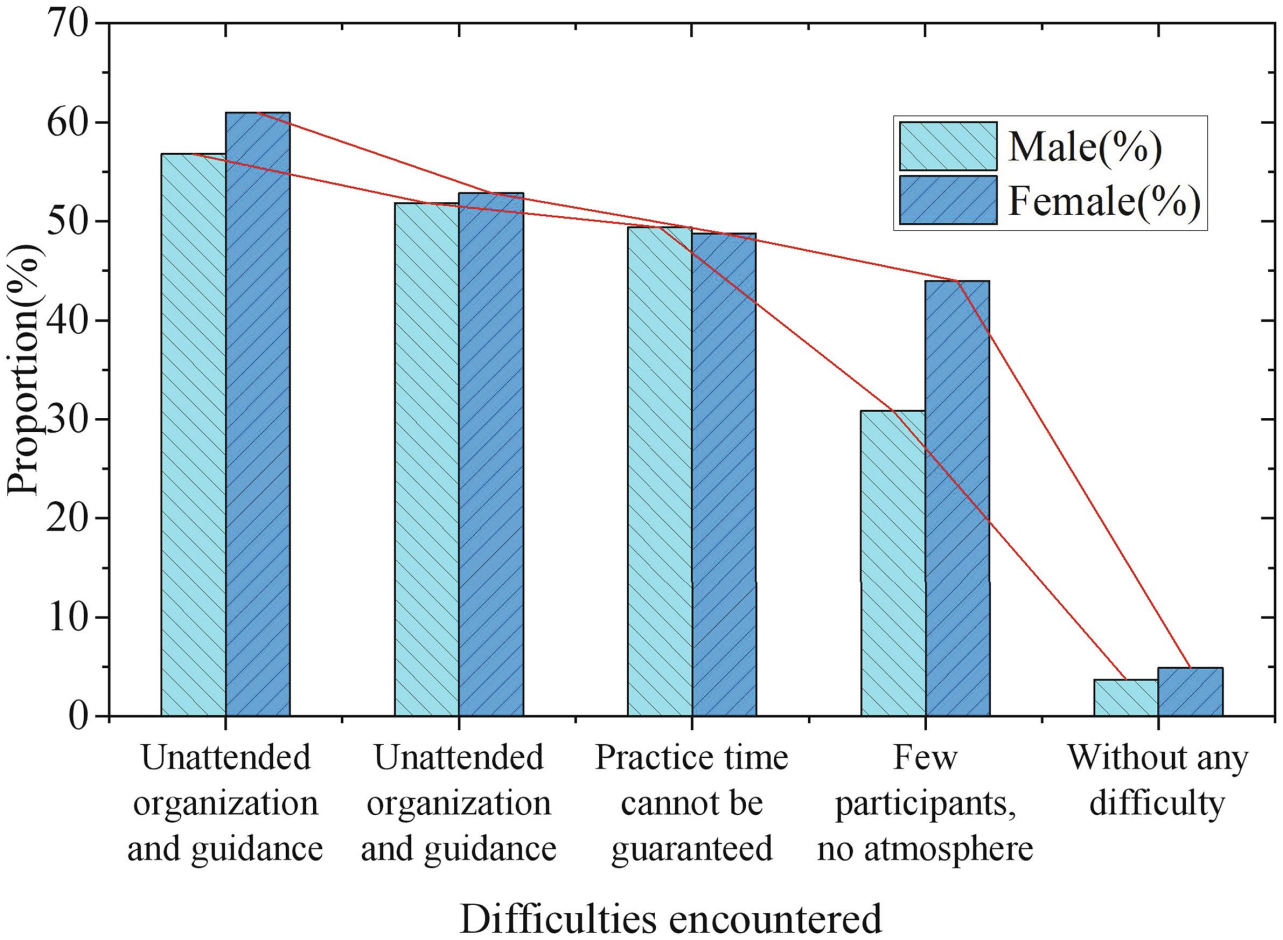
The development status of Guozhuang dance in universities (by gender).

**Figure 6:**
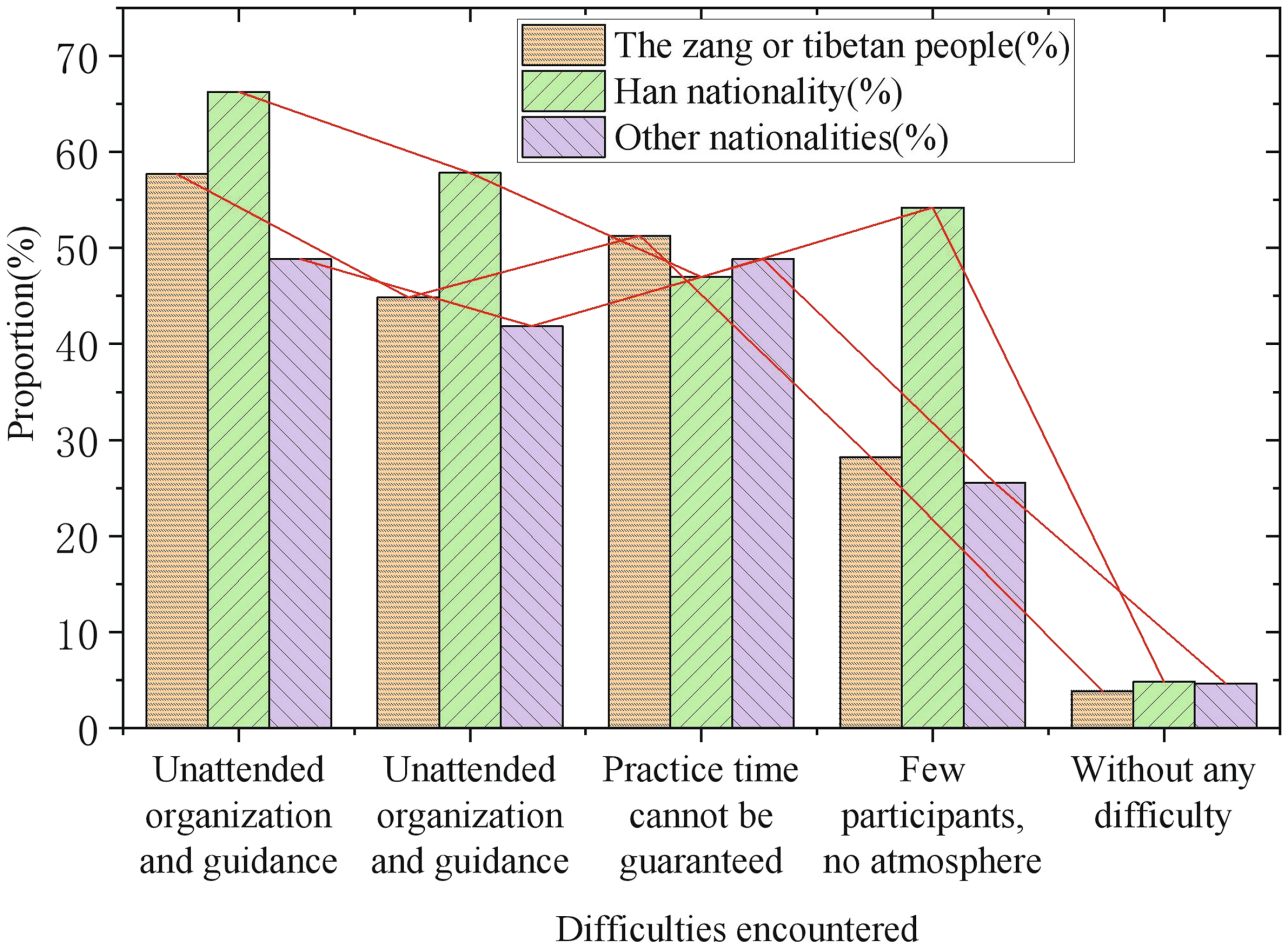
The development status of Guozhuang dance in universities (by ethnic).

As can be seen from Figure 4, most students have some understanding of the dance and music of Guozhuang dance, among which the proportion of boys is higher than that of girls, and graduate students have a deeper understanding of the dance than undergraduates. Among them, 54.55% of the students indicated that they knew this dance and music very well. As far as understanding is concerned, Tibetan students naturally understand their own dance better than Han and other ethnic minorities. The survey on the development status of the dance in this university is demonstrated in Figures 5, 6.

Figures 5, 6 illustrate that although many students are very willing to participate in the Guozhuang dance group, most of the students feel that there is no organization, lack of guidance, and poor music and dance foundation. The survey respondents of different genders, different educational backgrounds, and different ethnic groups encountered similar difficulties, showing that participation in the folk art of Guozhuang dance and its music has similar obstacles, which are very unfavorable to the spread of traditional art among college students.

### 4.2. The value impact of folk music and dance on the mental health of college students

**Figure 7:**
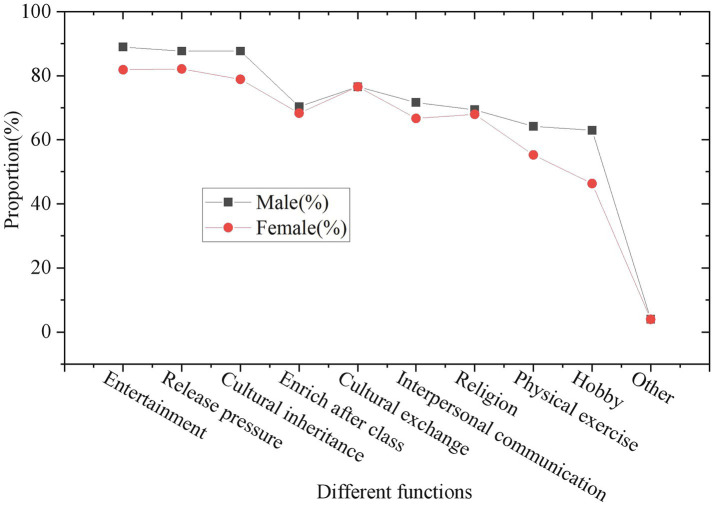
Student’s functional cognition survey on folk music and dance (statistics by gender).

**Figure 8:**
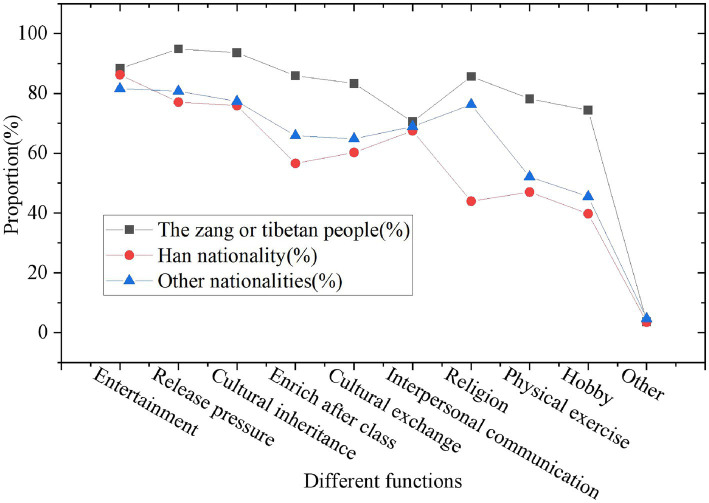
Student’s functional cognition survey on folk music and dance (statistics by ethnic).

The students’ functional cognition survey of folk music and dance is exhibited in Figures 7, 8.

**Figure 9:**
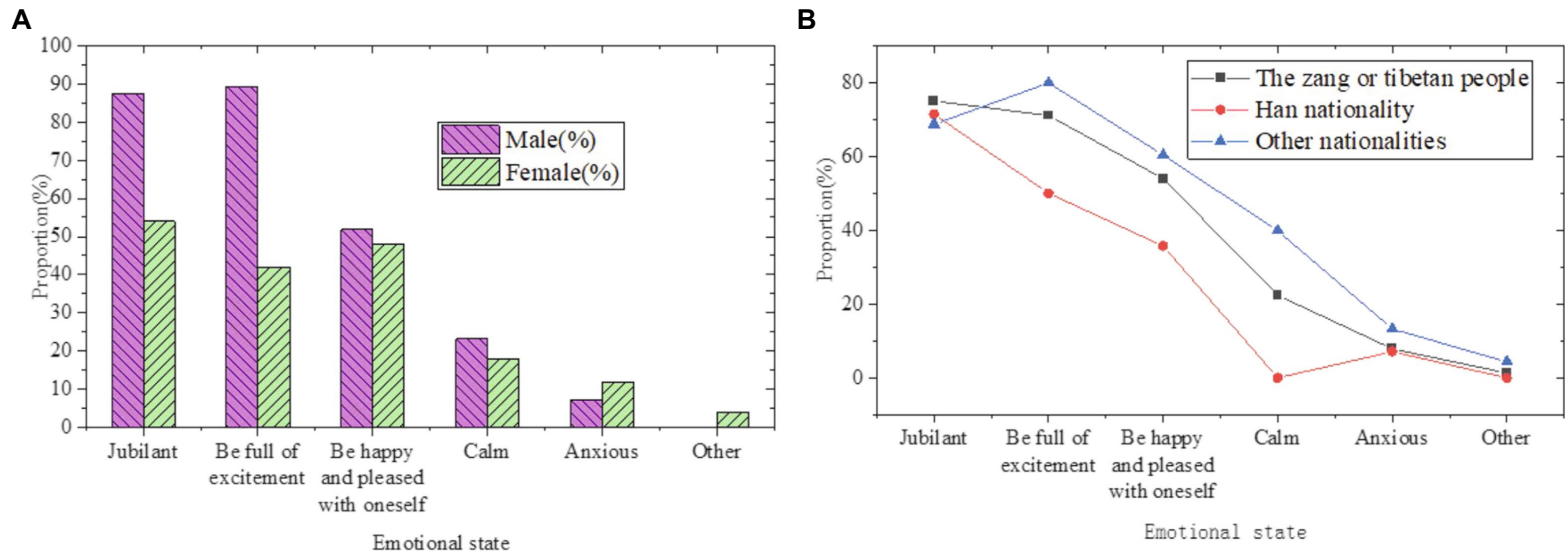
Statistics on the emotional status of students during the participation of Guozhuang dance and folk art (**A**: by gender; **B**: by educational background).

**Figure 10:**
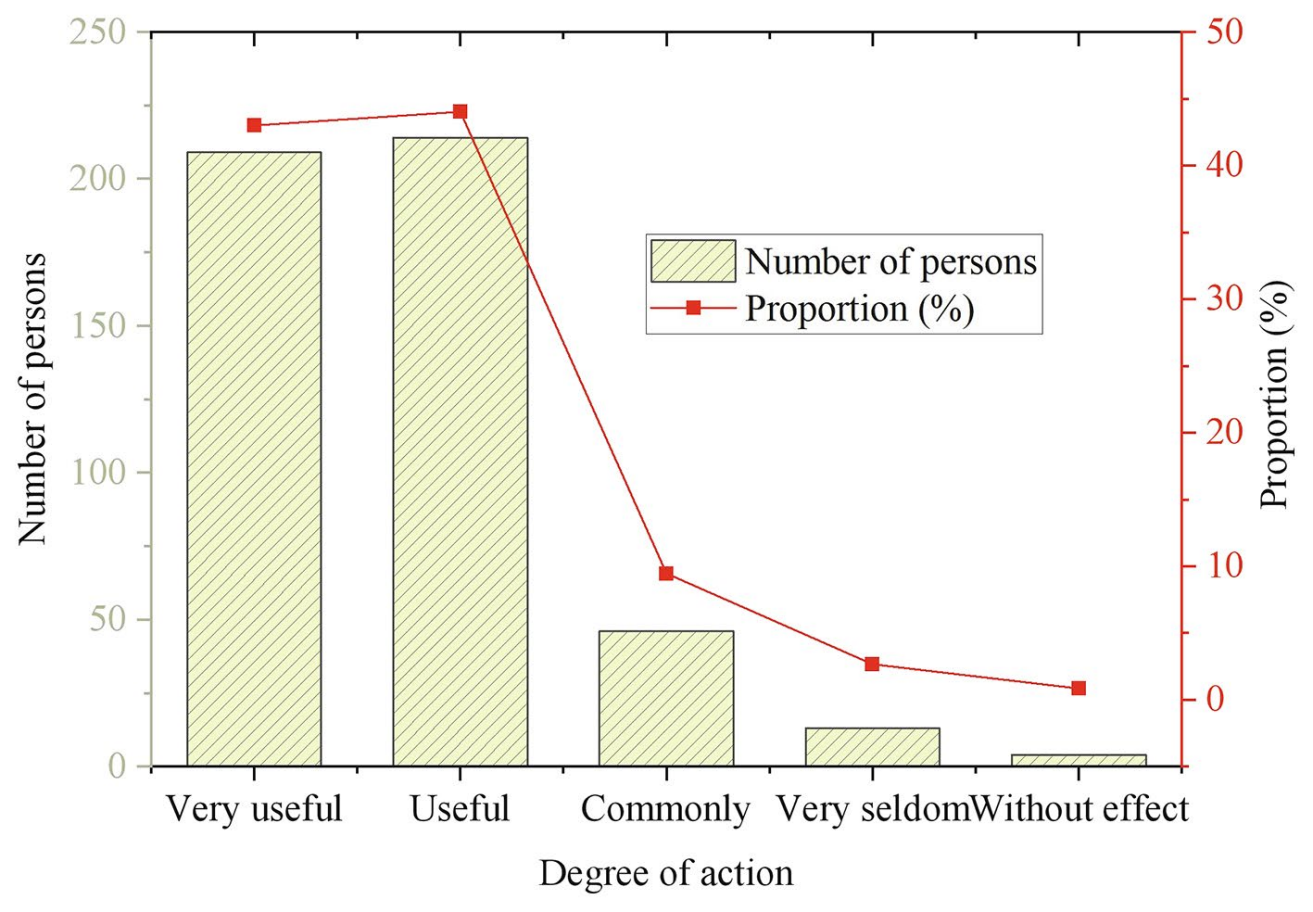
Can the participation of folk art play a role in regulating emotions or releasing stress?

**Figure 11:**
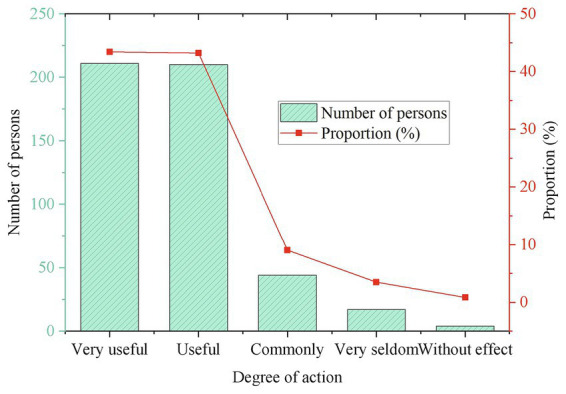
Whether the process of participation in folk art has a role in the development of mental health.

Figures 7, 8 shows that most of the surveyed students believe that Guozhuang dance and its music have more functions, especially in entertaining the public, relieving stress, regulating emotions and spreading national culture, and the degree of recognition is above 80%. Compared with the Han nationality and other ethnic minorities, the Tibetan students have a relatively high degree of recognition of the functions of this dance. On the one hand, it is the culture and art of their own ethnic group, so they naturally understand and love it better. On the other hand, it also reflects the importance of folk music to cultural dissemination. During the performance, Guozhuang Dance is not only a form of dance and music, but also integrates the Tibetan cultural heritage with rich cultural connotations. The process of students learning dance is also the process of disseminating ICH. Music and dance have a very important positive effect on the enhancement of human physical and psychological functions, and can also play a good role in promoting mental health. The value of promoting physical and mental health is gradually reflected in the process of students learning dance and music. The specific survey results of folk art on the value of mental health are illustrated in Figures 9–11.

In Figure 9, most of the students are in a happy mood when participating in the Guozhuang dance. Among the students, 71.7% say they are in high spirits, 66.98% believe they are in high spirits, 50% feel they are happy and content, and only 9.43% think they are anxious during dancing and listening to music. It means that in the process of participating in this folk art, the self-emotion of the students can be satisfied, which is similar to the students of different nationalities and genders.

In Figure 10, it can be found that 41.8% of the students think it is very useful for emotion regulation and stress relief, and 46.31% feel it is useful in the process of participating in this kind of folk art. It indicates that more than 80% of the students believe that the cheerful music rhythm and enthusiastic dance form of Guozhuang Dance can help relieve mental unhappiness to a certain extent.

Figure 11 refers that the proportion of students who consider the folk art of Guozhuang dance very useful for the development of mental health is 36.95, and 49.75% think it is useful. It shows that a total of 86.7% of the students believe that this dance is helpful to the development of students’ mental health. It can be seen that the relaxed and happy music rhythm of this dance in the school enables students to relax their minds and body, relieve stress and effectively help students relieve mental health problems such as depression and anxiety during the participation process.

## 5. Discussion

From the above research, it can be found that the special folk music dance music of the Guozhuang dance is very effective in the development of mental health. This is inseparable from the theory of music psychotherapy, and it also expands the scope of repertoire in music therapy. This is similar to the conclusions in the study. Lee and Lee (43) aimed to determine the effect of Korean folk music therapy programs on emotional behavior and interpersonal function in patients with schizophrenia. The levels of emotional behavior and interpersonal function in the experimental group were significantly higher than those in the control group, indicating that the Korean folk music therapy regimen was an effective intervention. Winter Plumb et al. (44) described the progress of a study subject receiving goal-focused positive psychotherapy over multiple sessions, capturing the subtleties and responsiveness of the theory. As a result, in addition to reduced symptoms, clients gained more agency, clarity of ethical values, genuine concern for others, interaction with a wider range of feelings, and self-compassion. Anderson et al. (45) investigated the role of gender and personality in predicting the musical preferences of college undergraduates. They evaluated students’ responses using the Big Five Personality Scale and a Short Test Scale of Music Preferences. Based on the results, they recommended that therapists assessed the patient’s personality before assigning them to music therapy. Richards (46) noted that the World Tourism Organization reaffirmed that cultural tourism was an important part of international tourism consumption, accounting for more than 39% of the number of tourists. The study of cultural tourism has also developed rapidly, particularly in the areas of cultural consumption, cultural motivation, heritage preservation, the economics of cultural tourism, and anthropology and its relationship with the creative economy. Therefore, the role of music therapy in psychological problems is very obvious. Meanwhile, other factors, such as the type of music, the surrounding cultural environment, and the patient’s personality, need to be considered.

### 5.1. Exploration of the development path of folk music’s ICH

From the above survey, it can be found that the value of the ICH of folk music and dance to college students also reflects the importance of the physical and mental development of contemporary young people. As an important component of ICH, folk music contains the performance of various ethnic groups in daily life and social activities. It is a characteristic folk music culture with diverse forms and rich connotations, and it is also a very vital part of Chinese folk art. Folk music is not only a traditional culture, but also contains many values in ethnology, folklore, cultural studies, human progress, and artistic esthetics. And by virtue of its regional characteristics and musical rhythm close to the soul, it plays a crucial role in people’s physical and mental health development. Therefore, it is particularly important to safeguard and inherit the ICH of folk music. However, in the information age, people’s esthetic awareness, lifestyle, and economic structure have undergone different changes. As a result, the cultural inheritance of folk music is faced with many problems, such as the crisis of inheritors, the low level of public attention to folk music culture, the lack of publicity, and the lack of corresponding policies and regulations. In particular, young people either know little or are not interested in it, and have little interest in learning folk music with local feelings, sacrificial beliefs, and ancient legends, all of which affect the survival and safeguarding of the ICH of folk music. It is extremely complex and arduous to realize the sustainable development of folk music’s ICH. From the perspectives of people’s ideas, overall system reform, and safeguarding methods, it explores the path of its development.

1. The education of ICH of folk music in various parts of China has been strengthened, and groups of all ages are encouraged to actively participate.

**Figure 12:**
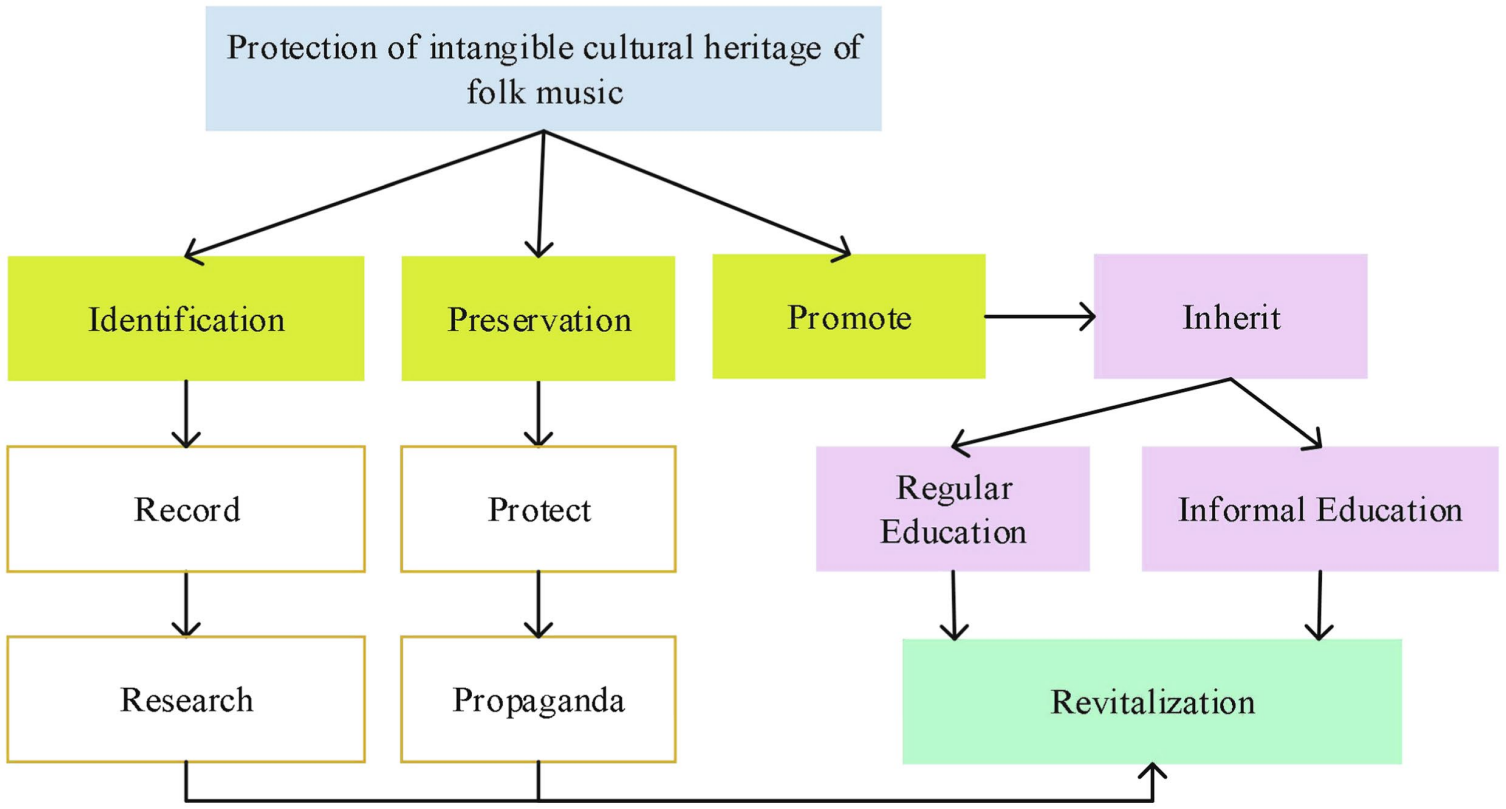
Ways to safeguard the ICH of folk music.

Because this kind of material culture originates from the folk, the key safeguarding work should also be placed on the folk, inherited from the folk, strengthen local education, social education, and school education, and carry out the safeguarding of ICH jointly by various parties. Figure 12 displays the specific content.

In all regions of the country, especially in the classrooms of colleges, and primary and secondary schools in provinces with many ethnic minorities, the ICH education of folk music has been popularized. Folk music is included in the music education classroom, and outstanding folk music inheritors are hired to teach irregularly in the classroom to increase students’ understanding and participation in folk music culture. College students are encouraged to conduct surveys on folk music, increase such elective courses, and cultivate their interest in folk music culture.

2. The government’s support is increased, and a diversified safeguarding system is established.

The safeguarding of ICH of folk music is inseparable from sound laws and regulations. At present, China has few laws and regulations specifically for folk music, so such a policy is needed to ensure that the inheritance of folk music’s ICH has evidence to follow and policy guarantees. In addition, it is necessary to provide financial subsidies and key support to its inheritors, especially the endangered music types, which need to be paid attention to. In the process of declaration and review of the ICH of ethnic minority folk music, efforts are being made to increase the proportion of the ICH of ethnic minorities in the overall heritage. The construction of public infrastructure required for the display of folk music’s ICH should also be improved, including inheritance and training areas, exhibition areas, collection areas, and performance areas.

3. The team-building of inheritors of the ICH of folk music is strengthened.

The inheritor is the foundation of the safeguarding of ICH. Folk music is lively and dynamic. The safeguarding of it requires not only the preservation of relevant images, text materials, creation facilities, etc., but also the inheritance of the spirit. It is a significant direction for the inheritance of folk music to establish a team of inheritors with complete structure, a reasonable team, and a clear hierarchy.

4. The digital system of the ICH of folk music is improved.

The development of computer technology has given birth to digital technologies represented by information technology, storage technology, scanning and printing technology, etc. With the help of digital means, it can help the classification and large-capacity storage of folk music-related information, which can improve the work efficiency of cultural staff in collecting and studying ICH.

5. The industrialization of the intangible culture of folk music has been vigorously promoted to increase its influence.

The relationship between culture and tourism is inherently mutually reinforcing. Folk music cultural resources are of great significance to the growth of tourism for the ICH projects. Combining folk music projects with the development of the cultural industry, developing rationally based on safeguarding, and taking the route of attracting tourists from other places and expanding music culture tourism can not only bring economic benefits to areas with relatively backward economic levels, but also publicize folk music.

## 6. Conclusion

There are many ethnic minorities in China, and the excellent folk music culture contained in them is not only a treasure left by history, but also the content that must be studied for future generations to inherit music culture. Nevertheless, the status quo shows that many ICHs of folk music have not been well safeguarded and inherited, and some are even in danger of being lost. Now all parts of the country are focusing on the development of cultural and tourism integration. By studying how to reasonably develop the ICH of folk music as a tourism project under this background, for one thing, it promotes folk music and for another develops tourism, which brings economic benefits to the local area. Furthermore, because of its local and national characteristics, folk music is different from ordinary popular music. The unique music rhythm, dance movements, and special musical instruments can bring people a certain psychological therapeutic effect. Tibetan Guozhuang dance is taken as the object, and a questionnaire survey is carried out on the college students of a university for nationalities. The purpose is to analyze the students’ awareness and liking of the dance, as well as the value of the dance in mental health, emotional regulation, and stress relief. It turned out that most of the students do not understand this kind of folk art. More than 80% of the students consider that in the process of participating in this dance, accompanied by a cheerful and clear music rhythm, it is very useful to relieve emotional tension, anxiety, stress, and other mental health problems. It denotes that this research has played a great role in the safeguarding of folk art. However, there are still some shortcomings. For example, in the process of the questionnaire survey, a folk art survey is selected. The results of the data may be a bit one-sided, targeting only the student population. In the future, it is planned to carry out surveys on the status quo of the ICH of folk music among social groups, to propose more perfect and effective safeguarding measures.

## Data availability statement

The original contributions presented in the study are included in the article/supplementary material, further inquiries can be directed to the corresponding author.

## Author contributions

The author confirms being the sole contributor of this work and has approved it for publication.

## Funding

This work was supported by Ministry of Education in China, Project of Humanities and Social Sciences (no. 21YJC760055).

## Conflict of interest

The author declares that the research was conducted in the absence of any commercial or financial relationships that could be construed as a potential conflict of interest.

## Publisher’s note

All claims expressed in this article are solely those of the authors and do not necessarily represent those of their affiliated organizations, or those of the publisher, the editors and the reviewers. Any product that may be evaluated in this article, or claim that may be made by its manufacturer, is not guaranteed or endorsed by the publisher.

## References

[1] Yan WJ Li KR. Sustainable Cultural Innovation Practice: Heritage Education in Universities and Creative Inheritance of Intangible Cultural Heritage Craft. Sustainability. (2023) 15:1194. doi: 10.3390/su15021194

[2] Jørgensen MT McKercher B. Sustainability and integration–the principal challenges to tourism and tourism research. J Travel Tour Mark. (2019) 36:905–16. doi: 10.1080/10548408.2019.1657054

[3] Sun J Ling L Huang ZJ. Tourism migrant workers: the internal integration from urban to rural destinations. Ann Tour Res. (2020) 84:102972. doi: 10.1016/j.annals.2020.102972

[4] Harris R. “A weekly Mäshräp to tackle extremism”: music-making in Uyghur communities and intangible cultural heritage in China. Ethnomusicology. (2020) 64:23–55. doi: 10.5406/ethnomusicology.64.1.0023

[5] Lee J. Promoting majority culture and excluding external ethnic influences: China’s strategy for the UNESCO ‘intangible’cultural heritage list. Social Identities. (2020) 26:61–76. doi: 10.1080/13504630.2019.1677223

[6] Shrivastava P Smith MS O’Brien K. Transforming sustainability science to generate positive social and environmental change globally. One Earth. (2020) 2:329–340. doi: 10.1016/j.oneear.2020.04.010 33501419 PMC7181980

[7] Mac Con Iomaire M. Recognizing food as part of Ireland’s intangible cultural heritage. Folk Life. (2018) 56:93–115. doi: 10.1080/04308778.2018150240202

[8] Jeffery L Rotter R. Safeguarding sega: transmission, inscription, and appropriation of Chagossian intangible cultural heritage. Int J Herit Stud. (2019) 25:1020–33. doi: 10.1080/13527258.2018.1555671

[9] Su J. Managing intangible cultural heritage in the context of tourism: Chinese officials’ perspectives. J Tour Cult Chang. (2020) 18:164–86. doi: 10.1080/14766825.2019.1604720

[10] Stepputat K Kienreich W Dick CS. Digital methods in intangible cultural heritage research: a case study in tango Argentino. J Comput Cult Heritage. (2019) 12:1–22. doi: 10.1145/3279951

[11] Nakku VB Agbola FW Miles MP. The interrelationship between SME government support programs, entrepreneurial orientation, and performance: A developing economy perspective. J Small Business Manag. (2020) 58:2–31. doi: 10.1080/00472778.2019.1659671

[12] Zhou Y Sun J Huang Y. The digital preservation of intangible cultural heritage in China: a survey. Preserv Digit Technol Cult. (2019) 48:95–103. doi: 10.1515/pdtc-2019-0004

[13] Stegemann T Geretsegger M Phan Quoc E Riedl H Smetana M. Music therapy and other music-based interventions in pediatric health care: an overview. Medicines. (2019) 6:25. doi: 10.3390/medicines6010025, PMID: 30769834 PMC6473587

[14] de Witte M Pinho AS Stams GJ Moonen X Bos AER van Hooren S. Music therapy for stress reduction: a systematic review and meta-analysis. Health Psychol Rev. (2022) 16:134–59. doi: 10.1080/17437199.2020.1846580, PMID: 33176590

[15] Devlin K Alshaikh JT Pantelyat A. Music therapy and music-based interventions for movement disorders. Curr Neurol Neurosci Rep. (2019) 19:83. doi: 10.1007/s11910-019-1005-0 31720865

[16] Partesotti E Peñalba A Manzolli J. Digital instruments and their uses in music therapy. Nord J Music Ther. (2018) 27:399–418. doi: 10.1080/08098131.2018.1490919

[17] Lam HL Li WTV Laher I Wong RY. Effects of music therapy on patients with dementia—A systematic review. Geriatrics. (2020) 5:62. doi: 10.3390/geriatrics5040062, PMID: 32992767 PMC7709645

[18] Golino AJ Leone R Gollenberg A Christopher C Stanger D Davis TM. Impact of an active music therapy intervention on intensive care patients. Am J Crit Care. (2019) 28:48–55. doi: 10.4037/ajcc2019792, PMID: 30600227

[19] Zeppegno P Krengli M Ferrante D. Psychotherapy with music intervention improves anxiety, depression and the redox status in breast cancer patients undergoing radiotherapy: a randomized controlled clinical trial. Cancers. (2021) 13:1752. doi: 10.3390/cancers13081752 33916933 PMC8067630

[20] Moreno-Morales C Calero R Moreno-Morales P Pintado C. Music therapy in the treatment of dementia: a systematic review and meta-analysis. Front Med. (2020) 7:160. doi: 10.3389/fmed.2020.00160, PMID: 32509790 PMC7248378

[21] Gramaglia C Gambaro E Vecchi C Licandro D Raina G Pisani C. Outcomes of music therapy interventions in cancer patients—a review of the literature. Crit Rev Oncol Hematol. (2019) 138:241–54. doi: 10.1016/j.critrevonc.2019.04.004, PMID: 31121392

[22] Hadley S Thomas N. Critical humanism in music therapy: imagining the possibilities. Music Ther Perspect. (2018) 36:168–74. doi: 10.1093/mtp/miy015

[23] de la Rubia Ortí JE García-Pardo MP Iranzo CC JJC M Castillo SS Rochina MJ. Does music therapy improve anxiety and depression in alzheimer's patients? J Altern Complement Med. (2018) 24:33–6. doi: 10.1089/acm.2016.0346 28714736

[24] Su J. Understanding the changing intangible cultural heritage in tourism commodification: the music players’ perspective from Lijiang, China. J Tour Cult Chang. (2019) 17:247–68. doi: 10.1080/14766825.2018.1427102

[25] Masoud H Mortazavi M Farsani NT. A study on tourists' tendency towards intangible cultural heritage as an attraction (case study: Isfahan, Iran). City Cult Soc. (2019) 17:54–60. doi: 10.1016/j.ccs.2018.11.001

[26] Beck W. The folksongs of Jharkhand: an intangible cultural heritage of tribal India. Int J Humanit Social Sci. (2018) 11:763–6. doi: 10.5281/zenodo.1316740

[27] Su J. A difficult integration of authenticity and intangible cultural heritage?: the case of Yunnan, China. China Perspect. (2021) 3:29–39.

[28] Lv Z Han Y Singh AK Manogaran G Lv H. Trustworthiness in industrial IoT systems based on artificial intelligence. IEEE Trans Ind Inf. (2020) 17:1496–504. doi: 10.1109/TII.2020.2994747

[29] Xie S Yu Z Lv Z. Multi-disease prediction based on deep learning: a survey. Comput Model Eng Sci. (2021) 128:489–522. doi: 10.32604/cmes.2021.016728

[30] Perrot S. The apotropaic function of music inside the sanctuaries of Asklepios: ritual soundscape and votive offerings. Greek Roman Musical Stud. (2016) 4:209–30. doi: 10.1163/22129758-12341276

[31] Elpus K Abril CR. Who enrolls in high school music? A national profile of US students, 2009–2013. J Res Music Educ. (2019) 67:323–338. doi: 10.1177/0022429419862837

[32] Abrams B. Understanding humanistic dimensions of music therapy: editorial introduction. Music Ther Perspect. (2018) 36:139–43. doi: 10.1093/mtp/miy019

[33] Vaudreuil R Langston DG Magee WL Betts D Kass S Levy C. Implementing music therapy through telehealth: considerations for military populations. Disabil Rehabil Assist Technol. (2022) 17:201–10. doi: 10.1080/17483107.2020.1775312, PMID: 32608282

[34] Sharda M Silani G Specht K Tillmann J Nater U Gold C. Music therapy for children with autism: investigating social behaviour through music. Lancet Child Adolescent Health. (2019) 3:759–61. doi: 10.1016/S2352-4642(19)30265-2, PMID: 31494080

[35] Hardi T Kupi M Ocskay G Szemerédi E. Examining cross-border cultural tourism as an indicator of territorial integration across the Slovak–Hungarian border. Sustainability. (2021) 13:7225. doi: 10.3390/su13137225

[36] Tzima S Styliaras G Bassounas A. Harnessing the potential of storytelling and mobile technology in intangible cultural heritage: A case study in early childhood education in sustainability. Sustainability. (2020) 12:9416. doi: 10.3390/su12229416

[37] Syafrini D Fadhil Nurdin M Sugandi YS Miko A. The impact of multiethnic cultural tourism in an Indonesian former mining city. Tour Recreat Res. (2020) 45:511–25. doi: 10.1080/02508281.2020.1757208

[38] Liu X Li M. Safeguarding intangible cultural heritage to promote mental healthcare in China: challenges to maintaining the sustainability of safeguarding efforts. Int J Soc Psychiatry. (2020) 66:311–3. doi: 10.1177/0020764020904752, PMID: 32009504

[39] Ranwa R. Impact of tourism on intangible cultural heritage: case of Kalbeliyas from Rajasthan, India. J Tour Cult Chang. (2022) 20:20–36. doi: 10.1080/14766825.2021.1900208

[40] Sibel SÜ. The function of Turku bars (Turkish folk music bars) in promotion of Turkish culture and its role in cultural heritage transmission as A recreation business. OPUS Int J Soc Res. (2020) 15:1625–48. doi: 10.26466/opus.681384

[41] Qiu Q Zhang M. Using content analysis to probe the cognitive image of intangible cultural heritage tourism: an exploration of Chinese social media. ISPRS Int J Geo Inf. (2021) 10:240. doi: 10.3390/ijgi10040240

[42] Kim Y. The early beginnings of Nordoff-Robbins music therapy. J Music Ther. (2004) 41:321–39. doi: 10.1093/jmt/41.4.321, PMID: 15762836

[43] Lee KJ Lee K. Effect of Korean folk music intervention on schizophrenia inpatients' emotional behavior and interpersonal relationship functioning. Arch Psychiatr Nurs. (2020) 34:115–21. doi: 10.1016/j.apnu.2020.02.002, PMID: 32513460

[44] Winter Plumb EI Hawley KJ Conoley CW Scheel MJ. From cosmetics to compassion: A case study of goal focused positive psychotherapy. Psychotherapy. (2020) 57:414–25. doi: 10.1037/pst0000287, PMID: 31999190

[45] Anderson I Gil S Gibson C Wolf S Shapiro W Semerci O. “Just the way you are”: Linking music listening on Spotify and personality. Soc Psychol Person Sci. (2021) 12:561–572. doi: 10.1177/1948550620923

[46] Richards G. Cultural tourism: A review of recent research and trends. J Hosp Tour Manag. (2018) 36:12–21. doi: 10.1016/j.jhtm.2018.03.005

